# Hybrid histone deacetylase-kinase inhibitor potentiates venetoclax-induced cell death in chronic lymphocytic leukemia

**DOI:** 10.1016/j.htct.2025.103757

**Published:** 2025-04-02

**Authors:** Anali Del Milagro Bernabe Garnique, Jorge Antonio Elias Godoy Carlos, Natalia Sudan Parducci, Mauricio Temotheo Tavares, Karoline de Barros Waitman, Keli Lima, Leticia Veras Costa-Lotufo, Roberto Parise-Filho, João Agostinho Machado-Neto

**Affiliations:** a Department of Pharmacology, Institute of Biomedical Sciences, University of São Paulo, São Paulo, SP, Brazil; b Department of Pharmacy, Faculty of Pharmaceutical Science, University of São Paulo, São Paulo, SP, Brazil; c Department of Cancer Biology, Dana-Farber Cancer Institute, Boston, MA, United States; d Department of Biological Chemistry and Molecular Pharmacology, Harvard Medical School, Boston, MA, United States; e Laboratory of Medical Investigation in Pathogenesis and Targeted Therapy in Onco-Immuno-Hematology (LIM-31), Department of Internal Medicine, Hematology Division, Faculty of Medicine, University of São Paulo, São Paulo, SP, Brazil

Dear Editor,

Chronic lymphocytic leukemia (CLL) is characterized by the abnormal production of mature B lymphocytes in the blood, bone marrow, spleen, and lymphoid tissues. However, these cells are dysfunctional due to genomic alterations. CLL cells express functional B-cell receptors (BCRs) on their surface and can be classified into two subgroups based on somatic hypermutations in the variable regions of the *immunoglobulin heavy chain* (*IGHV*) genes. CLL patients with somatic mutations in the *IGHV* gene (M-CLL) generally show better survival rates than those with the unmutated *IGHV* gene (UM-CLL). Clinically, CLL typically presents with lymphocytosis, along with lymphadenopathy or cytopenias (anemia, thrombocytopenia, and neutropenia).^1^ BCR signaling is essential in CLL, with Bruton's tyrosine kinase (BTK) playing a key role. BTK inhibitors (BTKis) block this signaling by binding to BTK, thereby hindering the proliferation and survival of both malignant and normal B cells. BTK is crucial for activating survival pathways such as nuclear factor kappa B (NFκB) and mitogen-activated protein kinase (MAPK).^2^

Over the past three decades, several drugs have been approved, including combination chemotherapies and immunotherapies, such as fludarabine, cyclophosphamide, and rituximab, as well as chlorambucil (CLB) combined with obinutuzumab. More recently, inhibitors targeting key pathways have emerged, such as ibrutinib (Bruton's tyrosine kinase inhibitor), idelalisib (PI3Kδ inhibitor), and venetoclax (BCL2 inhibitor).^1^^,^^3^ Despite these diverse treatment options, genetic abnormalities associated with chemoresistance frequently arise in CLL patients, leading to the use of immunotherapy as a first-line treatment.^3^ Additionally, resistance to fludarabine (flu-refractory) remains a major cause of treatment failure in CLL.^4^ Therefore, the development of new therapeutic agents for CLL treatment is crucial.

Venetoclax (ABT-199/GDC-0199) is a highly selective BCL2 inhibitor that mimics the BH3 protein by competitively binding to the anti-apoptotic BCL2 protein. This action releases BAK and BAX, subsequently inducing apoptosis.^5^ CLL cells exhibit constitutively high expression of BCL2, an anti-apoptotic protein that renders them resistant to cell death. This resistance contributes to the accumulation of long-lived, clonal lymphocytes characteristic of the disease.^6^ This feature makes BCL2 inhibitors promising targets for chemotherapy. Venetoclax became a Food and Drug Administration (FDA)-approved standard treatment in June 2018 as a second-line therapy for CLL patients, demonstrating deep and durable responses, regardless of adverse prognostic features such as a 17p deletion.^7^

Histone modification modulates chromatin structure and gene expression, with abnormal histone acetylation linked to cancer development. The histone function is regulated by multiple post-translational modifications, including the reversible acetylation of ε-amino groups of histone's lysine. Histone acetylation is tightly controlled by a balance between histone acetyltransferases (HATs) and histone deacetylases (HDACs).^8^

Vorinostat, the first FDA-approved HDAC inhibitor for lymphoma, is now also used clinically for other cancers.^9^ Elevated HDAC activity in CLL B-cells is associated with shorter treatment-free and overall survival, serving as an independent prognostic marker for overall survival and refining the accuracy of established prognostic factors.^10^ Preclinical studies show that depsipeptide (FR901228), suberoylanilide hydroxamic acid (SAHA or vorinostat), and chidamide inhibit cellular processes critical to CLL progression and chemoresistance by targeting HDAC activity.^11^^,^^12^^,^^13^ Due to the lack of selectivity and toxicity associated with certain HDAC inhibitors, there is a pressing need for selective inhibitors targeting specific HDAC classes, underscoring the importance of studying novel HDAC inhibitors. However, some novel class I HDAC inhibitors tested in CLL patients as monotherapy presented limited clinical efficacy^14^, suggesting that its combination with other therapies could be a strategy to improve efficacy, while avoiding undesirable side effects.

Anilino-purine-benzohydroxamate hybrids were synthesized as dual inhibitors targeting kinases and HDACs. Among these, compound 4d showed promising potency and specificity against leukemia and lymphoma. Notably, some of the identified kinase targets, including BTK, JAK2, and JAK3, are of particular interest in CLL.^15^ In the present study, we characterized the cellular and molecular effects of 4d and evaluated its combination with venetoclax in a CLL cell model.

The mRNA expression data for HDACs from healthy donors (normal B cells; n = 20) and CLL patients (n = 103) were sourced from the publicly accessible AmaZonia! Database 2008.^16^ MEC-1 cells were kindly provided by Prof. Rodrigo Alexandre Panepucci (Hemocenter of Ribeirão Preto, Brazil) and cultured according to the recommendations of Deutsche Sammlung von Mikroorganismen und Zellkulturen GmbH (DSMZ). Compound 4d was synthesized as previously described.^15^ Vorinostat was obtained from Sigma-Aldrich (St. Louis, MO, USA). The structures of HDAC/kinase inhibitors are shown in Figure 1. Cellular and molecular assays were performed as previously described.^17^ In summary, cell viability was assessed by MTT assay, clonogenic potential by colony formation assay in methylcellulose (MethoCult 4230; StemCell Technologies Inc., Vancouver, BC, Canada), apoptosis by annexin V/propidium iodide (PI) staining followed by flow cytometry, cell cycle analysis using PI staining was used for assess DNA content and flow cytometry, protein expression and activation by Western blot with specific antibodies (Supplementary Table 1), and gene expression by quantitative PCR with specific primers (Supplementary Table 2). Statistical analyses were performed using GraphPad Prism 8 (GraphPad Software Inc.), with the Mann-Whitney test, analysis of variance (ANOVA) and Bonferroni post-test, or Student's t-test used as appropriate. A *p*-value <0.05 was considered statistically significant.

**Figure 1:**
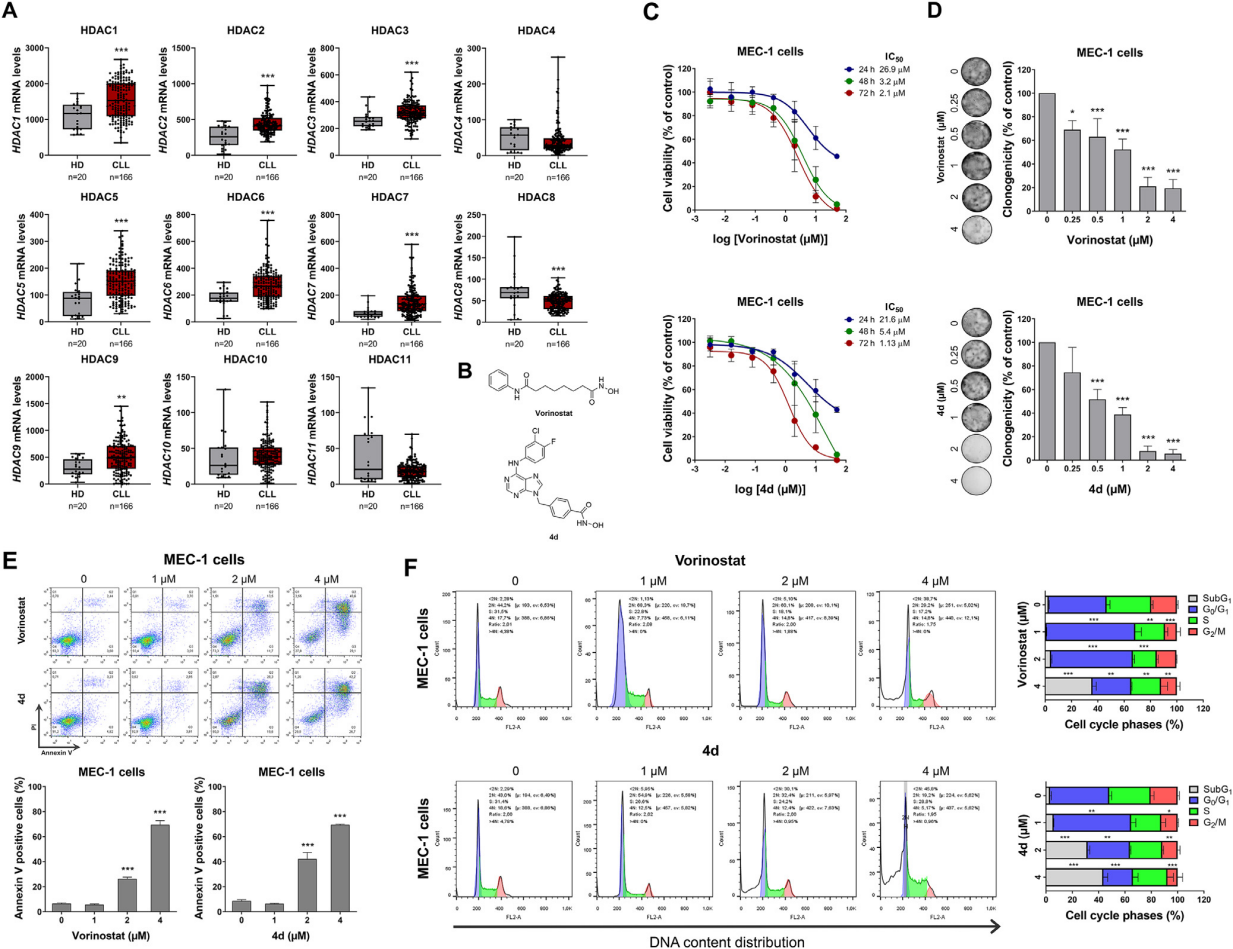
Histone deacetylases (HDACs) are highly expressed and a potential druggable target in chronic lymphocytic leukemia.

(A) The mRNA levels of HDACs (1-11) were measured in samples from healthy donors (n = 20) and CLL patients (n = 166) using data from the Amazonia! database (2008). Gene expression data on the Y-axis were derived from cDNA microarray analysis performed with Affymetrix HGU133 Plus 2.0 arrays. Datasets were cross-referenced using tumor-specific identification numbers, and the sample size for each group is indicated. ^⁎⁎^*p*-value <0.01 and ^⁎⁎⁎^*p*-value <0.0001; Mann-Whitney test. (B) The chemical structure of the HDAC inhibitors used are illustrated. (C) Dose- and time-dependent cytotoxicity was assessed using the methylthiazolyl tetrazolium (MTT) assay in MEC-1 cells treated with either vehicle or increasing concentrations of vorinostat or compound 4d (0.0032–50 µM) for 24, 48, or 72 h. Cell viability was quantified as a percentage relative to vehicle-treated controls. Results are expressed as the mean ± SD from at least three independent experiments. The IC_50_ values for each compound are provided in the corresponding Figure. (D) MEC-1 cells were cultured in semisolid medium with either vehicle or increasing concentrations of vorinostat or compound 4d. After 12 days, colonies containing viable cells were detected by adding MTT reagent. Representative colony images are shown, and bar graphs represent the mean ± SD from at least three independent experiments. Statistical significance was determined by ANOVA followed by Bonferroni post-test; *p-value <0.05, *p-value *<*0.001. (E) MEC-1 cells were labeled with APC-annexin V and propidium iodide (PI) after 48-h treatment with either vehicle or the specified concentrations of vorinostat or compound 4d. Representative dot plots are shown for each condition, with apoptotic cells (annexin V^+^ cells) identified in the upper and lower right quadrants (Q2 + Q3). Bar graphs display the mean ± SD from at least three independent experiments. The p-values and cell line are indicated in the graphs; *p-value *<*0.0001; ANOVA followed by Bonferroni post-test. **(**F) Cell cycle phases were assessed by analyzing DNA content through propidium iodide staining and flow cytometry after treating MEC-1 cells with either vehicle, vorinostat, or compound 4d at the specified concentrations for 48 h. Representative histograms are shown for each condition, with bar graphs presenting the mean ± SD from at least three independent experiments. *p*-values and cell lines are indicated in the graphs; *p-value <0.05, ^⁎⁎^*p*-value <0.01, *p-value *<*0.001; ANOVA followed by Bonferroni post-test.

The mRNA levels of *HDAC1, HDAC2, HDAC3, HDAC5, HDAC6, HDAC7,* and *HDAC9* were elevated in CLL patients compared to healthy donors (all p-value <0.05), while *HDAC8* expression was lower in CLL patients (Figure 1A). In MEC-1 cells, treatment with vorinostat and 4d reduced cell viability in a dose- and time-dependent manner (Figure 1C). Similarly, these compounds reduced clonal growth in a concentration-dependent manner (Figure 1D) and induced apoptosis (Figure 1E). Both vorinostat and 4d caused cell cycle arrest in the G0/G1 phase at lower concentrations, indicating a cytostatic effect, while higher concentrations led to an increase in the sub-G1 cell population, indicating a cytotoxic effect.

On a molecular level, both compounds strongly induced acetylation of histone H3 and alpha-tubulin, suggesting the inhibition of class I and II HDACs. However, only compound 4d slightly reduced ERK1/2 and NFkB phosphorylation, likely reflecting its hybrid activity on kinases (Figure 2A). Furthermore, markers of cell death such as PARP1 cleavage and γH2AX, were more prominently induced by 4d. Both compounds activated autophagic flux, as shown by SQSTM1/p62 degradation and/or LC3B consumption (Figure 2B). Exploratory gene analysis involving cell cycle progression, DNA damage, apoptosis, and autophagy showed a similar profile of impacted cellular and molecular processes for both compounds (Figure 2C).

**Figure 2:**
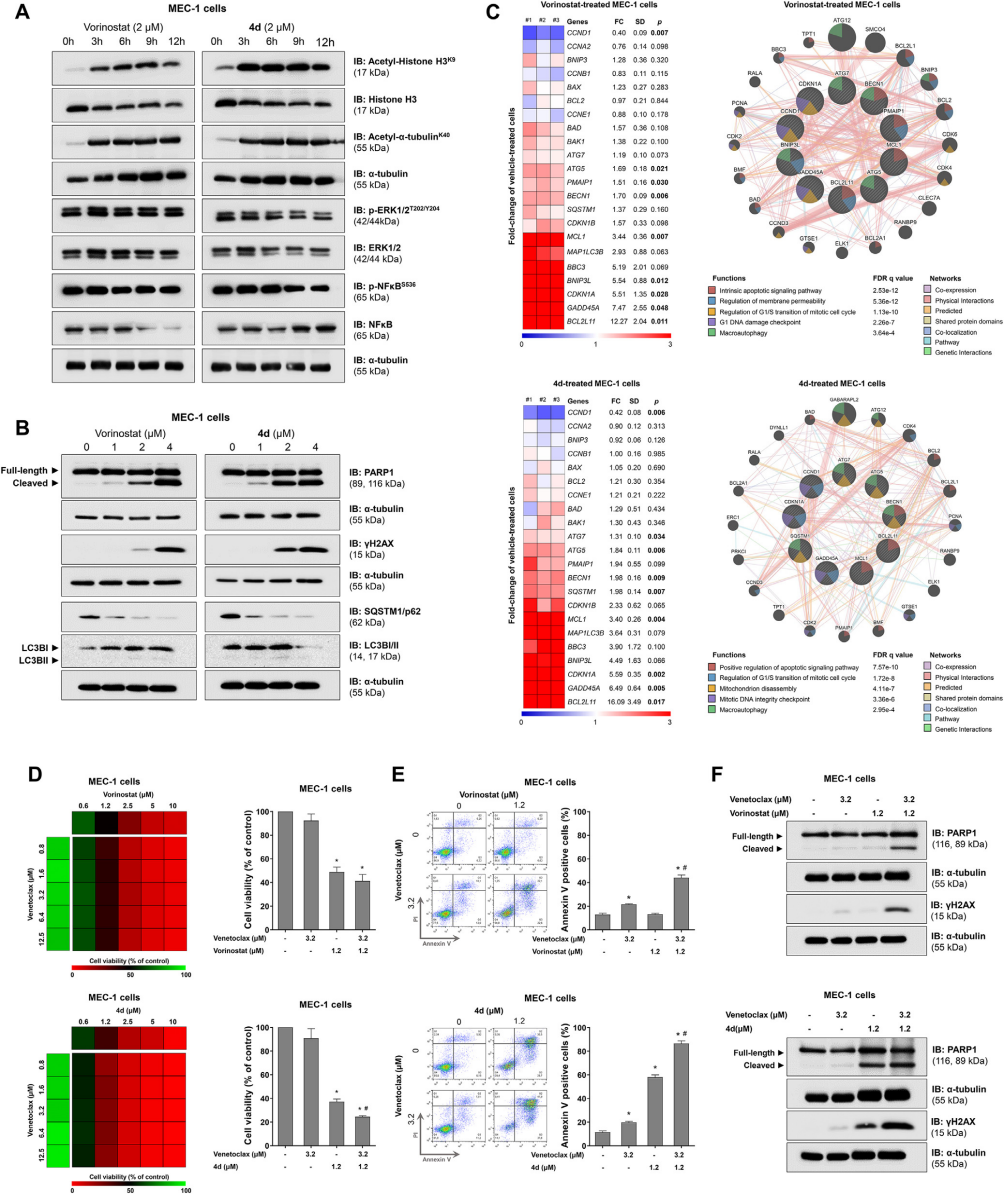
4d, a hybrid histone deacetylase (HDAC)-kinase inhibitor, shows greater efficacy in potentiating venetoclax-induced apoptosis in MEC-1 cells.

(A) Western blot analysis was conducted to detect acetyl-histone H3 (K9), histone H3, acetyl-α-tubulin (K40), α-tubulin, p-ERK1/2, ERK1/2, p-NFkB, and NFkB in total cell extracts from MEC-1 cells treated with vehicle, vorinostat (2 µM), or compound 4d (2 µM) for 0, 3, 6, 9, or 12 h. (B) Western blot analysis was conducted to detect total and cleaved PARP1, γH2AX, SQSTM1/p62, and LC3BI/II in total cell extracts from MEC-1 cells treated with vehicle, vorinostat (1, 2, and 4 µM), or compound 4d (1, 2, and 4 µM) for 24 h. Membranes were subsequently reprobed with antibodies against total protein or α-tubulin as loading controls and developed using the SuperSignal™ West Dura Extended Duration Substrate system with a G:Chemi XX6 imaging system. (C) The heatmap displays the gene expression profile of MEC-1 cells treated with vehicle, vorinostat (2 μM), or compound 4d (2 μM) for 24 h. Blue denotes reduced mRNA levels, while red denotes increased mRNA levels, normalized to vehicle-treated cells (n = 3). Fold-change (FC), standard deviation (SD), and *p*-values were calculated using Student's t-test. A gene network of vorinostat- or 4d-modulated genes was generated using the GeneMANIA database (https://genemania.org/). Genes with significant modulation are represented as crosshatched circles, while interacting genes added by the software are shown as non-crosshatched circles. The main biological interactions, associated functions, and false discovery rate (FDR) q-values are detailed in the Figure. (D) Dose-response cytotoxicity for the combinations of vorinostat plus venetoclax and compound 4d plus venetoclax was evaluated using the methylthiazolyldiphenyl-tetrazolium bromide (MTT) assay. MEC-1 cells were exposed to vehicle or increasing concentrations of venetoclax in combination with vorinostat or compound 4d alone or together for 48 h, as indicated. Cell viability values are expressed as a percentage relative to vehicle-treated controls, with bar graphs highlighting context-relevant combinations. *p-value *<*0.05, treatment versus vehicle; ^#^*p*-value <0.05, monotherapy versus combination therapy; ANOVA with Bonferroni post-test. Data represents the mean from at least three independent experiments. (E) For cell death analysis, MEC-1 cells were labeled with APC-annexin V and propidium iodide (PI) after treatment with vehicle, venetoclax, vorinostat, or compound 4d alone or in combination for 48 h. Representative dot plots are shown for each condition, with the upper and lower right quadrants (Q2 + Q3) cumulatively representing the cell death population (annexin V^+^ cells). Bar graphs display the mean ± SD from at least three independent experiments; *p-value <0.05, treatment versus vehicle; ^#^*p*-value <0.05, monotherapy versus combination therapy; ANOVA with Bonferroni post-test. (F) Western blot analysis was conducted to detect total and cleaved PARP1, γH2AX and α-tubulin in total cell extracts from MEC-1 cells treated with vehicle, venetoclax, vorinostat, or compound 4d alone or in combination for 24 h.

Finally, combination assays with venetoclax and either vorinostat or 4d highlighted the superiority of the HDAC-kinase hybrid inhibitor compared to vorinostat. Although both compounds enhanced venetoclax-induced apoptosis, 4d demonstrated greater efficacy in viability assays (Figure 2D), apoptosis induction (Figure 2E), and molecular analysis (Figure 2F).

In summary, CLL patients exhibit increased expression of various HDACs. Vorinostat and 4d reduced cell viability and induced apoptosis in a CLL cell model, with 4d showing higher efficacy in combination with venetoclax. Molecularly, both inhibited HDAC activity, and 4d had additional effects on ERK1/2 and NFkB pathways. These findings suggest that the hybrid compound 4d holds promise for more effective therapies in CLL, warranting further studies focused on its clinical potential and combination with BCL2 inhibitors.

## Conflicts of interest

The authors declare no competing interests.

## Appendix Supplementary materials

Image, application 1

Image, application 2
