# Supplementary material for: Hybrid histone deacetylase-kinase inhibitor potentiates venetoclax-induced cell death in chronic lymphocytic leukemia
Source: Hematol Transfus Cell Ther. 2025 Apr 2;47(2):103757. doi: 10.1016/j.htct.2025.103757 (PMC11999328; doi:10.1016/j.htct.2025.103757)
Supplement: Supplementary file 1 [file mmc1.docx]

| **Supplementary Table 1.** List of antibodies used in the Western blotting analysis. | | | |
| --- | --- | --- | --- |
| **Primary antibody** | **Species** | **Dilution** | **Catalogue** |
| Acetyl-Histone H3^K9^ | Rabbit | 1:1000 | #9649 / Cell signaling |
| Histone H3 | Rabbit | 1:2000 | #4499 / Cell signaling |
| Acetyl-α-Tubulin^K40^ | Rabbit | 1:2000 | #5335 / Cell signaling |
| α-Tubulin | Rabbit | 1:4000 | #2144 / Cell signaling |
| p-ERK1/2^T202/Y204^ | Rabbit | 1:1000 | #9101 / Cell signaling |
| ERK1/2 | Rabbit | 1:1000 | #9102 / Cell signaling |
| p-NFĸB^S536^ | Rabbit | 1:1000 | #3033 / Cell signaling |
| NFĸB | Rabbit | 1:1000 | #8242 / Cell signaling |
| PARP1 | Rabbit | 1:2000 | #9542 / Cell signaling |
| γH2AX | Rabbit | 1:4000 | #9718 / Cell signanling |
| SQSTM1/p62 | Mouse | 1:1000 | #88588 / Cell signaling |
| LC3BI/II | Rabbit | 1:2000 | #2775 / Cell signaling |
| Anti-mouse HRP | Rabbit | 1:4000 | #7076 / Cell signaling |
| Anti-rabbit HRP | Goat | 1:4000 | #7074 / Cell signaling |
