# Supplementary material for: Hybrid histone deacetylase-kinase inhibitor potentiates venetoclax-induced cell death in chronic lymphocytic leukemia
Source: Hematol Transfus Cell Ther. 2025 Apr 2;47(2):103757. doi: 10.1016/j.htct.2025.103757 (PMC11999328; doi:10.1016/j.htct.2025.103757)
Supplement: Supplementary file 2 [file mmc2.doc]

| **Supplementary Table 2.** Primer sequences and concentrations. | | |
| --- | --- | --- |
| **Gene^1^** | **Sequence** | **Concentration** |
| *ATG5* | FW: GGGCCATCAATCGGAAAC  RV: AGCCACAGGACGAAACAG | 300 nM |
| *ATG7* | FW: CGTTGCCCACAGCATCATCTTC  RV: TCCCATGCCTCCTTTCTGGTTC | 300 nM |
| *BAD* | FW: CACCAGCAGGAGCAGCCAAC  RV: CGACTCCGGATCTCCACAGC | 300 nM |
| *BAK1* | FW: TGAGTACTTCACCAAGATTGCCA  RV: AGTCAGGCCATGCTGGTAGAC | 300 nM |
| *BAX* | FW: GAGCTGCAGAGGATGATTGC  RV: CAGCTGCCACTCGGAAAA | 300 nM |
| *BBC3* | FW: GACCTCAACGCACAGTACGAG  RV: AGGAGTCCCATGATGAGATTG | 300 nM |
| *BCL2* | FW: ATGTGTGTGGAGAGCGTCAA  RV: ACAGTTCCACAAAGGCATCC | 300 nM |
| *BCL2L11* | FW: ATGTCTGACTCTGACTCTCG  RV: CCTTGTGGCTCTGTCTGTAG | 300 nM |
| *BECN1* | FW: TCTGAAGAGGACCTGGACCCT  RV: GGCTCACGTCCATCTCGTC | 300 nM |
| *BNIP3* | FW: ATATGGGATTGGTCAAGTCGG  RV: CGCTCGTGTTCCTCATGCT | 300 nM |
| *BNIP3L* | FW: ACACCAGCAGGGACCATAGC  RV: TTTCTTCAAAGCCTCGACTTCC | 300 nM |
| *CCNA2* | FW: GCCTTTCATTTAGCACTCTACA  RV: CAGGGTATATCCAGTCTTTCG | 300 nM |
| *CCNB1* | FW: GTCTCCATTATTGATCGGTTCATG  RV: CCAATTTCTGGAGGGTACATTTCT | 300 nM |
| *CCND1* | FW: CTCGGTGTCCTACTTCAAATG  RV: AGCGGTCCAGGTAGTTCAT | 300 nM |
| *CCNE1* | FW: TATATGGCGACACAAGAAAATG  RV: GTGCAACTTTGGAGGATAGA | 300 nM |
| *CDKN1A* | FW: TGTCACTGTCTTGTACCCTTGT  RV: GCCGGCGTTTGGAGTGGTAG | 300 nM |
| *CDKN1B* | FW: ACTCTGAGGACACGCATTTGGT  RV: TCTGTTCTGTTGGCTCTTTTGTT | 300 nM |
| *GADD45A* | FW: AAGGATGGATAAGGTGGGG  RV: CTGGATCAGGGTGAAGTGG | 300 nM |
| *MCL1* | FW: GTAATAACACCAGTACGGACGG  RV: TCCCGAAGGTACCGAGAGAT | 300 nM |
| *MAP1LC3B* | FW: AAGGCGCTTACAGCTCAATG  RV: CTGGGAGGCATAGACCATGT | 300 nM |
| *PMAIP1* | FW: CGCGCAAGAACGCTCAACC  RV: CACACTCGACTTCCAGCTCTGCT | 300 nM |
| *SQSTM1* | FW: TGAGGAACAGATGGAGTCGGATAA  RV: GGGACTGGAGTTCACCTGTAGACG | 300 nM |
| *ACTB* | FW: AGGCCAACCGCGAGAAG  RV: ACAGCCTGGATAGCAACGTACA | 150 nM |
| *HPRT1* | FW: GAACGTCTTGCTCGAGATGTGA  RV: TCCAGCAGGTCAGCAAAGAAT | 150 nM |

^1^Genes are reported according to Human Genome Organization (HUGO) Gene Nomenclature Committee (HGNC).

Abbreviations: FW, forward; RV, reverse.
